# The distributions of hematologic and biochemical values in healthy high-school adolescents in Japan

**DOI:** 10.1371/journal.pone.0242272

**Published:** 2020-11-17

**Authors:** Tatsuhiko Azegami, Tomoyasu Nishimura, Ayano Murai-Takeda, Nobuko Yamada-Goto, Yasunori Sato, Masaaki Mori

**Affiliations:** 1 Keio University Health Center, Yokohama-shi, Japan; 2 Department of Preventive Medicine and Public Health, Keio University School of Medicine, Tokyo, Japan; Oregon State University, UNITED STATES

## Abstract

Laboratory tests of adolescents are often interpreted by using reference intervals derived from adults, even though these populations differ in their physical and physiologic characteristics and disease susceptibility. Therefore, to examine the distribution of laboratory values specific for adolescents, we analyzed hematologic and biochemical measurements obtained from 12,023 healthy Japanese adolescents (ages 15 through 18 years; male, 9165; female, 2858) during 2009 through 2018. Distributions were shown as medians with 95% (2.5th and 97.5th percentiles) of values and were compared with those from previous studies that examined similar Asian populations. There were some differences between hematologic parameters, serum creatinine and uric acid concentration, and lipid levels of Japanese adults and adolescents. In comparison with other Asian populations, the distributions of serum uric acid and high-density–lipoprotein cholesterol in the present study were slightly higher than those in the other studies. Although further research is need, the distributions of hematologic and biochemical tests in adolescents may have the potential to facilitate the early identification and management of disease in this population.

## Introduction

Reference intervals play critical roles in the interpretation of laboratory parameters and enable disease risk assessment in asymptomatic people. Because adolescents are in the transitional phases of growth and development, they differ from children and adults in physical and physiologic characteristics and disease susceptibility. Despite previous studies that showed differences in laboratory reference intervals between adolescents and adults [1, 2], laboratory tests of adolescents typically are interpreted by using reference intervals derived from adult populations.

In general, the sooner reversible risk factors are corrected, the lower the likelihood of potential future disease [3]. Therefore, early identification of abnormal laboratory values will enable early intervention and benefit adolescents at high risk for various diseases. However, adequately assessing the risk of disease in adolescents requires laboratory reference intervals specific for this age group.

Establishing accurate and useful laboratory reference intervals for adolescents has been hampered by difficulty in recruiting a sufficiently large number of ‘healthy’ study subjects. Previous adolescent reference intervals from Japan are limited in utility because they were developed by using data from small numbers of hospitalized patients [4]. In addition, because students in Japan are not obligated to undergo routine hematology and clinical chemistry screening, laboratory data from healthy high-school adolescents are sparse.

In the current study, we analyzed the hematologic and biochemical parameters of more than 12,000 healthy Japanese high-school students to examine the distributions of laboratory values.

## Materials and methods

### Study design and participants

This study was approved by the Keio University School of Medicine Ethics Committee (Approved No. 2018–0253) and was conducted in accordance with the Declaration of Helsinki. According to the local ethical committee guidelines, written informed consent was waived, and opt-out methods was adopted to allow individuals who refuse to participant in the study to directly contact and indicate their intention to us.

This cross-sectional study included Japanese male and female high-school adolescents who ranged in age from 15 to 18 years and received annual medical checkups at the Keio University Health Center during 2009 through 2018. All participants were enrolled in 4 high schools in Tokyo and suburban Tokyo, Japan. Exclusion criteria for this study were: (1) body mass index (BMI) <17.0 kg/m^2^ or ≥25.0 kg/m^2^; (2) systolic blood pressure (BP) ≥140 mm Hg or diastolic BP ≥90 mm Hg; (3) maintenance medications for chronic diseases; (4) current pregnancy or within 1 year after childbirth; (5) hospitalization during the preceding 1 month; and (6) past or present medical history of heart disease, renal disease, diabetes mellitus, autoimmune disease, chronic infectious disease, or cancer.

### Anthropometric and biochemical measurements

Standing height and body weight were measured without shoes and outer clothing. BMI was calculated as body weight divided by the square of the height (kg/m^2^). BP was measured by a trained nurse using an electronic sphygmomanometer (BP-103i II, Omron Colin Co, Ltd., Tokyo, Japan) on the right arm of a seated participant. When the measured BP was ≥140/90 mm Hg (2009 through 2010) or ≥140/85 mm Hg (2011 through 2018), the BP was re-measured during that clinical visit, and the last measurement was used for the analysis.

Blood samples were collected under non-fasting conditions. For hematologic tests, peripheral blood cells were counted by using flow cytometry. For biochemical assays, serum creatinine was measured by using the enzymatic method, uric acid by the uricase/peroxidase method, and high-density–lipoprotein cholesterol (HDLC) and low-density–lipoprotein cholesterol (LDLC) by the direct method.

### Statistical analyses

Baseline characteristics were summarized as means (standard deviation [SD]) for continuous variables and as frequencies (percentages) for categorical variables. To capture the natural spread of values for each parameter in healthy adolescents, no outliers were deleted. The distributions were shown as medians with 95% (between the 2.5th and 97.5th percentiles) of value. In addition, means ± SD were determined for comparison with median values. All statistical analyses were performed by using SPSS software (version 25.0, IBM Corporation, Armonk, New York, USA).

## Results

**Fig 1:**
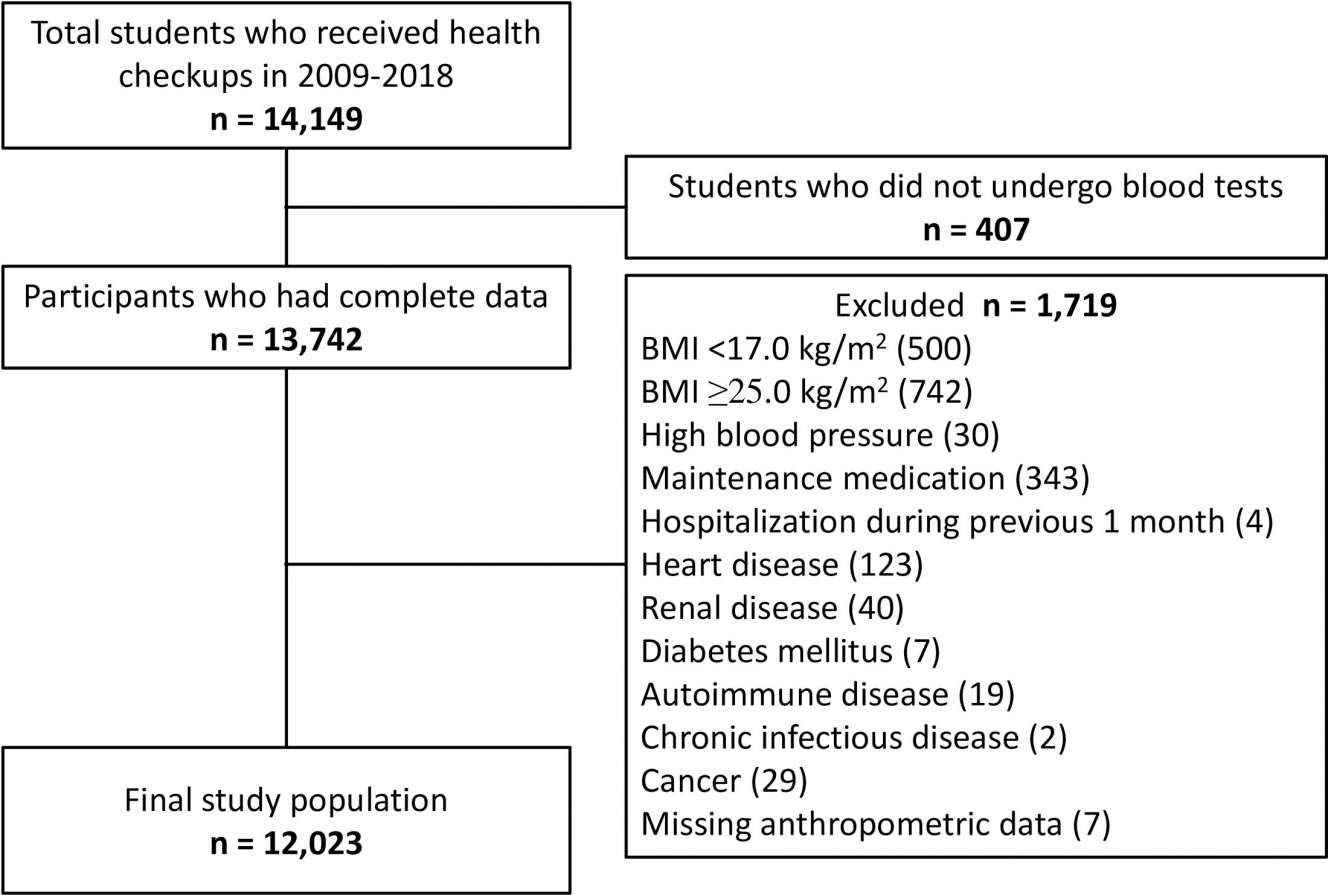
Flow chart for the participant selection.

A total of 14,149 adolescents received school-required health checkups during 2009 through 2018 (Fig 1). Of the 13,742 students that underwent hematology and clinical chemistry tests, 1719 persons (12.5%) were excluded because of BMI <17.0 kg/m^2^ (*n* = 500) or ≥25.0 kg/m^2^ (*n* = 742); high BP defined as systolic BP ≥140 mm Hg or diastolic BP ≥90 mm Hg (*n* = 30); maintenance medications for chronic diseases (*n* = 343); hospitalization during the previous 1 month (*n* = 4); past or present medical history of heart disease (*n* = 123), renal disease (*n* = 40), diabetes mellitus (*n* = 7), autoimmune disease (*n* = 19), chronic infectious disease (*n* = 2), or cancer (*n* = 29); and missing anthropometric data (*n* = 7). Of the 1719 persons excluded, 127 fulfilled more than one exclusion criterion.

The total 14,149 students received health checkups during 2009 through 2018. Because of incomplete data collection, 407 students were excluded. Of the 13,742 participants, 1,719 participants were excluded because of health conditions that can affect the results of hematologic and biochemical parameters.

The final analytical sample comprised 12,023 adolescents (male, 9165; female, 2858), and the characteristics of the study population are shown in Table 1. Almost all participants belonged to a single, cohesive ethnic group native to Japan; none of the participants used tobacco products or drank alcohol. Most (80.0%) of the male participants were 16 years old, whereas 91.2% of the female participants were 15 years old. Mean BMI was 20.6 (SD, 1.8) kg/m^2^ in male students and 19.9 (SD, 1.7) kg/m^2^ in female adolescents, respectively. Mean BPs were 113.0/62.1 (SD, 11.9/8.5) mm Hg in male subjects and 107.4/60.6 (SD, 11.9/8.1) mm Hg in female students.

**Table 1:** Characteristics of the study population.

	Male (*n* = 9165)	Female (*n* = 2858)
Age		
15 years, *n* (%)	934 (10.2)	2606 (91.2)
16 years, *n* (%)	7332 (80.0)	252 (8.8)
17 years, *n* (%)	883 (9.6)	0 (0.0)
18 years, *n* (%)	16 (0.2)	0 (0.0)
Height, cm, mean (SD)	170.8 (5.6)	158.6 (5.2)
Weight, kg, mean (SD)	60.1 (6.7)	50.1 (5.1)
Body mass index, kg/m^2^, mean (SD)	20.6 (1.8)	19.9 (1.7)
Systolic blood pressure, mm Hg, mean (SD)	113.0 (11.9)	107.4 (11.9)
Diastolic blood pressure, mm Hg, mean (SD)	62.1 (8.5)	60.6 (8.1)

Hematologic and biochemical parameters from our study population were stratified according to sex and are reported in Table 2 as median values with 95% (i.e., 2.5th through 97.5th percentile) of values or as mean ± SD, as appropriate. We also showed the median values and mean after the outlier removal based on 3SD methods in S1 Table. Because there is no consensus about whether to include or not outliers, we compared the values derived from data that include outliers (Table 2) and those from data that exclude ± 3SD outliers (S1 Table). However, there was little effect of excluding outliers. We then compared the data from our adolescent participants with the reference intervals from previous studies [4–8] that examined similar populations (Table 3).

**Table 2:** The distributions of hematologic and biochemical values in Japanese high-school adolescents, stratified according to sex.

	Male participants (*n* = 9165)		Female participants (*n* = 2858)	
Parameter	Median (95% interval)	Mean ± SD	Median (95% interval)	Mean ± SD
WBC, /μL	6300 (4000–10600)	6519 ± 1690	6100 (3900–9700)	6255 ± 1481
RBC, ×10^6^/μL	5.17 (4.57–5.82)	5.17 ± 0.32	4.59 (4.15–5.19)	4.60 ± 0.28
Hemoglobin, g/dL	15.2 (13.4–17.1)	15.2 ± 1.0	13.3 (10.9–15.0)	13.3 ± 1.0
Hematocrit, %	45.6 (40.4–50.5)	45.6 ± 2.6	40.9 (35.3–45.4)	40.8 ± 2.5
Platelets, ×10^3^/μL	24.9 (16.9–35.7)	25.3 ± 4.7	26.1 (18.0–37.7)	26.5 ± 5.0
Creatinine, mg/dL	0.80 (0.62–1.02)	0.81 ± 0.10	0.60 (0.45–0.80)	0.61 ± 0.09
Uric acid, mg/dL	5.90 (3.70–8.10)	5.91 ± 1.07	4.60 (3.00–6.60)	4.64 ± 0.88
HDLC, mg/dL	62.0 (42.0–89.0)	63.1 ± 11.9	68.0 (47.0–96.0)	68.6 ± 12.2
LDLC, mg/dL	89.0 (53.0–141.0)	90.4 ± 22.4	99.0 (59.0–155.6)	101.0 ± 24.9

HDLC, high-density–lipoprotein cholesterol; LDLC, low-density–lipoprotein cholesterol; RBC, red blood cells; WBC, white blood cells

**Table 3:** Comparison of reference intervals in the current and previous studies.

		This study			Tanaka T. et al. (4)			Bandesh K. et al. (5)			Li Y. et al. (6)			Kelishadi R. et al. (7)			Cho S.M. et al. (8)		
		Japanese			Japanese			Indian			Chinese			Iranian			Korean		
Parameter	Sex	95% interval	age	*n*	95% interval	age	*n*	95% interval	age	*n*	95% interval	age	*n*	95% interval	age	*n*	95% interval	age	*n*
WBC, /μL	male	4000–10600	16	9165	3800–9600	16													
	female	3900–9700	15	2858	3800–9400	15													
RBC, ×10^6^/μL	male	4.57–5.82	16	9165	4.28–5.65	16													
	female	4.15–5.19	15	2858	4.00–5.10	15													
Hemoglobin, g/dL	male	13.4–17.1	16	9165	12.8–16.7	16													
	female	10.9–15.0	15	2858	11.8–14.9	15													
Hematocrit, %	male	40.4–50.5	16	9165	36.6–48.5	16													
	female	35.3–45.4	15	2858	35.0–43.6	15													
Platelets, ×10^3^/μL	male	16.9–35.7	16	9165	17.0–40.0	16													
	female	18.0–37.7	15	2858	17.0–41.0	15													
Creatinine, mg/dL	male	0.62–1.02	16	9165	0.45–0.98	16		0.29–0.89	11–17	2296	0.49–1.03	16	151	1.1–1.6	15–19	147	0.45–0.98	13–16	188
	female	0.45–0.80	15	2858	0.35–0.75	15		0.25–0.79	11–17	2534	0.47–1.02	15	143	1.1–1.6	15–19	147	0.37–0.72	9–14	752
Uric acid, mg/dL	male	3.70–8.10	16	9165	3.70–7.65	16		2.52–7.91	11–17	2419	2.99–6.59	16	153				3.0–7.6	11–16	591
	female	3.00–6.60	15	2858	2.90–6.35	15		2.03–6.13	11–17	2645	3.58–6.49	15	140				2.5–5.9	2–14	1168
HDLC, mg/dL	male	42.0–89.0	16	9165				30.2–70.0	11–17	3099				31–72	16–19	1153			
	female	47.0–96.0	15	2858				30.2–72.7	11–17	3903				31–72	16–19	1153			
LDLC, mg/dL	male	53.0–141.0	16	9165				44.5–140.4	11–17	3100				9.27–172.20	9–17	3545			
	female	59.0–155.6	15	2858				45.2–143.9	11–17	3900				9.27–172.20	9–17	3545			

HDLC, high-density–lipoprotein cholesterol; LDLC, low-density–lipoprotein cholesterol; RBC, red blood cells; WBC, white blood cells

Empty cells indicate that the data were unavailable. To convert from SI units to conventional units, SI units were divided by the following conversion factors: creatinine, 88.4; uric acid, 59.48; HDLC and LDLC, 0.02586.

## Discussion

In the present study, we focused on examining the distribution of hematologic and biochemical parameters in Japanese high-school adolescents. In Japan, high-school students range in age from 15 through 18 years, which is a crucial period for the prevention of non-communicable diseases [3]. Hematologic and biochemical examination in adolescents is an essential tool for early identification of preventable risk factors for and diagnosis of various diseases. For example, a deranged complete blood count often is the first clue of hematologic malignancies and anemia [9]; serum creatinine is commonly used to assess renal function and detect chronic kidney disease [10]; serum uric acid is sometimes useful to reveal hidden asymptomatic conditions including malignant disorders, hereditary diseases, and drug side effects [11]; and elevated levels of HDLC and LDLC in adolescents are frequently the first indicators of genetic dyslipidemias, such as familial hypercholesterolemia [12].

Consistent with adolescence as a transitional phase of growth and development between childhood and adulthood, hematologic and biochemical parameters differ somewhat between adolescents and adults [1, 2]. Compared with our current results for Japanese high-schoolers, Japanese adults have lower WBC, RBC, and platelet counts; lower creatinine and uric acid concentrations, and higher LDLC values [13]. Therefore, to detect hematologic and biochemistry abnormalities in adolescents, reference intervals specific to this age group should be established.

During the last decade, several studies have reported adolescent reference intervals for various routine biochemical parameters, in countries other than Japan. These efforts include the MoYo (Motivating Young people to maintain a healthy life-style) project in Austria [14]; the combined COPENHAGEN Puberty study and the Falun project in Denmark and Sweden [15]; the CSCC (Canadian Society of Clinical Chemistry) [1], CALIPER (Canadian Laboratory Initiative on Pediatric Reference Intervals) [16], and CHMS (Canadian Health Measures Survey) [17] projects in Canada; CASPIAN (Childhood and Adolescent Surveillance and Prevention of Adult Non-communicable diseases)–III in Iran [7]; KICoS (Kisumu Incidence Cohort Study) in western Kenya [18]; and other studies conducted in India [5], China [6], Korea [8], and Canada [19]. However, the reference intervals defined for creatinine, uric acid, HDLC, and LDLC, in particular, vary widely among studies, perhaps due at least in part to racial or ethnic differences [6]. Among Asian populations, the reference ranges for creatinine and uric acid in Chinese adolescents are closer to those for Korean high-school students [6]. Moreover, the height and weight of Japanese [20] and other Asian [6] adolescents are generally much lower than those of their counterparts who live in the United States [21] and Europe [22]. Therefore, in consideration of the possible confounding effect of ethnicity, we compared our current results with those of previous studies that examined Asian populations [4–8] (Table 3).

In the present study, the distribution of serum creatinine in male high-schoolers (0.62–1.02 mg/dL) was similar to those in other Japanese [4], Chinese [6], and Korean [8] studies; higher than that in an Indian study [5]; and lower than that in an Iranian study [7]. The distribution that we report here for female Japanese adolescents (0.45–0.80 mg/dL) was almost equal to that in a Chinese study [6]; slightly higher than those in other Japanese [4], Indian [5], and Korean [8] studies; and considerably lower than that in an Iranian study [7]. The main reason for these differences may be age. The previous Japanese study showed a progressive increase in serum creatinine until age 20 years in male adolescents and until age 15 years in female students [4], and the age range and creatinine values of the participants in the Iranian study [7] were greater than those for all other studies [4–6, 8], including our own. In addition, although the Jaffe method (used in the Korean study) is prone to overestimating serum creatinine concentration when compared with the enzymatic method [23], the measurement procedure did not seem overall to influence creatinine reference intervals among the various studies.

The distributions of serum uric acid that we obtained here (male, 3.7–8.1 mg/dL; female, 3.0–6.6 mg/dL) were slightly higher than those in the other studies. Although little information regarding factors that influence serum uric acid concentrations in adolescents is available, increased childhood BMI and low birth weight seem to be associated with increased serum uric acid level [24, 25]. However, because neither the present study nor those we compared included any data regarding childhood BMI or birth weight, the influence of these factors on the differing uric acid levels is unclear. In contrast, increased current BMI is clearly associated with increased serum uric acid [5]. In this regard, the mean BMIs of the participants in the present study (male, 20.6 kg/m^2^; female, 19.9 kg/m^2^) were slightly higher than those in the Chinese study (male, 20.2 kg/m^2^; female, 19.2 kg/m^2^) [6]. Therefore, although there is no direct evidence, it is plausible that differences in BMI contribute to the differences in uric acid levels among studies.

The HDLC levels in our Japanese adolescents were higher than those in two comparable studies [5, 7], whereas LDLC levels were equivalent among all three studies. Age does not appear to contribute to inter-study differences in HLDC levels, because serum HDLC remained consistent regardless of age among 6- to 17-year-old Indian children and adolescents [26]. According to a study in Brazilian children and adolescents, a low HDLC level is associated with paternal tobacco use, high levels of C-reactive protein, and a high triceps-to-subscapular index [27]. We did not assess these parameters, and this may be one of the limitations of the present study.

Although there are some strengths such as a large sample size and recruiting ‘healthy’ study subjects in the present study, our study has several limitations. First, because eligibility was restricted to Japanese high-school adolescents living in an urban area, our findings may not be generalizable to other racial and ethnic groups and different socioeconomic groups. However, because the anthropometric data of our study are comparable to those obtained from a Japanese national survey [20], our current results may be generalizable to other Japanese adolescents. Second, we did not obtain any family history, birth information, or childhood health information—all of which might have influenced the laboratory data we collected. Third, we were unable to obtain other biochemical parameters—including triglycerides, glucose, and liver enzymes—that are risk and diagnostic factors of dyslipidemia, diabetes mellitus, and non-alcoholic fatty liver disease.

In conclusion, the distribution of hematologic and biochemical values of Japanese adolescents were different from those of Japanese adults and those of other Asian adolescents. Examining the distributions of hematologic and biochemical parameters in adolescents will potential to contribute to the early identification and prevention of lifestyle-related diseases and merits future research.

## Supporting information

S1 Table The distributions of hematologic and biochemical values after the outlier removal. (DOCX)
