# Supplementary material for: The distributions of hematologic and biochemical values in healthy high-school adolescents in Japan
Source: PLoS One. 2020 Nov 17;15(11):e0242272. doi: 10.1371/journal.pone.0242272 (PMC7671557; doi:10.1371/journal.pone.0242272)
Supplement: S1 Table — (DOCX) [file pone.0242272.s001.docx]

S1 Table. The distributions of hematologic and biochemical values after the outlier removal.

|  | Male | |  | Female (n=2858) | |  |
| --- | --- | --- | --- | --- | --- | --- |
| Parameter | Median (95% interval) | Mean ± SD | n | Median (95% interval) | Mean ± SD | n |
| WBC, /μL | 6200 (4000-10000) | 6443 ± 1544 | 9055 | 6100 (3900-9300) | 6195 ± 1370 | 2823 |
| RBC, 10^6^/μL | 5.17 (4.57-5.81) | 5.17 ± 0.31 | 9132 | 4.58 (4.08-5.16) | 4.59 ± 0.28 | 2841 |
| Hemoglobin, g/dL | 15.2 (13.5-17.1) | 15.3 ± 0.9 | 9097 | 13.4 (11.4-15.0) | 13.3 ± 0.9 | 2811 |
| Hematocrit, % | 45.6 (40.7-50.4) | 45.6 ± 2.5 | 9111 | 40.9 (36.0-45.4) | 40.9 ± 2.3 | 2828 |
| Platelet, 10^3^/μL | 24.9 (17.0-35.1) | 25.2 ± 4.5 | 9098 | 26.1 (18.1-36.8) | 26.4 ± 4.7 | 2829 |
| Creatinine, mg/dL | 0.80 (0.62-1.00) | 0.81 ± 0.10 | 9109 | 0.60 (0.45-0.79) | 0.60 ± 0.08 | 2833 |
| Uric acid, mg/dL | 5.90 (3.70-8.00) | 5.90 ± 1.03 | 9114 | 4.60 (3.00-6.50) | 4.64 ± 0.85 | 2841 |
| HDLC, mg/dL | 62.0 (42.0-88.0) | 62.9 ± 11.5 | 9114 | 68.0 (47.0-94.0) | 68.4 ± 11.8 | 2841 |
| LDLC, mg/dL | 88.0 (53.0-137.0) | 89.8 ± 21.0 | 9089 | 99.0 (59.0-150.0) | 100.1 ± 23.1 | 2827 |

HDLC, high-density–lipoprotein cholesterol; LDLC, low-density–lipoprotein cholesterol; RBC, red blood cells; WBC, white blood cells
